# Is There Evidence for Aetiologically Distinct Subgroups of Idiopathic Congenital Talipes Equinovarus? A Case-Only Study and Pedigree Analysis

**DOI:** 10.1371/journal.pone.0017895

**Published:** 2011-04-20

**Authors:** Amanda H. Cardy, Linda Sharp, Nicola Torrance, Raoul C. Hennekam, Zosia Miedzybrodzka

**Affiliations:** 1 Clubfoot Research Group, University of Aberdeen, Aberdeen, Scotland; 2 National Cancer Registry Ireland, Cork, Ireland; 3 Department of Pediatrics, University of Amsterdam, Amsterdam, The Netherlands; Tor Vergata University of Rome, Italy

## Abstract

**Background:**

Idiopathic congenital talipes equinovarus (CTEV) is a common developmental foot disorder, the aetiology of which remains largely unknown. Some aspects of the epidemiology suggest the possibility of aetiologically distinct subgroups. Previous studies consider CTEV as a homogenous entity which may conceal risk factors in particular subgroups. We investigate evidence for aetiologically distinct subgroups of CTEV.

**Methods:**

Parents of 785 probands completed a postal questionnaire. Family pedigrees were compiled by telephone. Case-only analysis was used to investigate interactions between risk factors and sex of the proband, CTEV laterality and CTEV family history.

**Results:**

The male∶female ratio was 2.3∶1, 58% of probands were affected bilaterally and 11% had a first-second degree family history. There were modest interactions between family history and twin births (multivariate case - only odds ratio [ORca] = 3.87, 95%CI 1.19–12.62) and family history and maternal use of folic acid supplements in early pregnancy (ORca = 0.62, 95%CI 0.38–1.01); and between sex of the proband and maternal alcohol consumption during pregnancy (female, positive history and alcohol consumed: ORca = 0.33, 95%CI 0.12–0.89). Previous reports of an interaction between maternal smoking and family history were not confirmed. Relatives of female probands were affected more often than relatives of male probands.

**Conclusions:**

These results provide tentative evidence for aetiologically distinct CTEV subgroups. They support the ‘Carter effect’, suggesting CTEV develops though a multifactorial threshold model with females requiring a higher risk factor ‘load’, and suggest areas where future aetiological investigation might focus. Large multi-centre studies are needed to further advance understanding of this common condition.

## Introduction

Congenital talipes equinovarus (CTEV) is a common developmental disorder with birth prevalence of 1–4.5 per 1000.[1] Affected feet are inclined inwards, axially rotated outwards, and point downwards, with concomitant soft tissue abnormalities.[2] Severity ranges from cases that resolve with manipulation to those requiring multiple operations with disability and discomfort persisting into later life. Although some cases occur with other neuromuscular and neurological disorders, most affected children have idiopathic CTEV.[3]

Mechanical, neurological, muscular, bony, connective tissue and vascular mechanisms for idiopathic CTEV have all been proposed.[3] Although genetic and lifestyle/environmental factors are thought to be aetiologically relevant, the genetic model is unclear and little is known about non-genetic risk factors.[3] However, some aspects of the epidemiology suggest areas worthy of further study: twice as many males as females are affected[4]–[7] and there is evidence of the ‘Carter effect’ (higher risk in relatives of affected females);[8], [9] 7–21%[10], [11] of families report CTEV in first-degree relatives, and one study suggests that family history modifies the association between CTEV and maternal smoking;[10] around half of affected children have bilateral CTEV[1], [4], [12], [13] and mouse studies suggest the number of affected feet is a marker for genetic load.[14] These observations raise the possibility of aetiologically distinct CTEV subgroups. Previous studies have considered idiopathic CTEV as a homogenous entity that may have concealed risk factors relevant, or more important, in particular subgroups.

The ECCE (Exploring Causes of Clubfoot in Europe) study comprises the largest reported series of idiopathic CTEV with primary data collection. Here, we investigate interactions between epidemiological risk factors and family history, the proband's sex, and laterality of the condition. We also report family pedigree analyses.

## Methods

### Ethics Statement

The Grampian Research Ethics Committee approved the study and written consent was obtained from each participating family (most often the mother signed on behalf of her partner and participating children).

### Subjects

Subjects were recruited May 2001–May 2003 through two support groups, steps[15] in the United Kingdom and VOK[16] in the Netherlands. The support groups approached families by mail on behalf of the investigators. A parent of the affected child (generally the mother) completed a questionnaire that included: nature of the condition (laterality, treatment, other medical conditions), maternal reproductive history, parental lifestyle (tobacco, alcohol, folic acid supplement and oral contraceptive [OC] use in the periconceptional period of the index pregnancy), and CTEV family history. On questionnaire receipt, a clinical geneticist (ZM) reviewed details of the foot defect and any additional conditions to exclude syndromic cases and non-CTEV conditions. Pedigrees were elicited by telephone from families who reported CTEV in family members other than the proband.

### Statistical analysis

The analysis included unrelated index children with idiopathic CTEV. Case-only methods[17], [18] were used to investigate whether CTEV risk factors differed by presence/absence of CTEV family history; sex of the proband; or laterality of the condition. Analysis contrasted sub-groups of cases with particular combinations of these “stratification variables” and risk factor exposures (e.g. male/female proband and maternal folic acid use/non-use), with the “association” between the stratification variable and risk factor (strictly the interaction, or departure from a multiplicative relationship) expressed as a case-only odds ratio (ORca). The stratification variables reference categories were: no family history; male; and unilateral CTEV. The primary analysis concerned first or second-degree family history. Using logistic regression, a “minimally adjusted” ORca was computed for each risk factor adjusted for country. Factors where the likelihood ratio test (LRT) p value was ≤0.1 in minimally adjusted analysis were considered for inclusion in multivariate models. Final multivariate models included country and variables where p≤0.1 for the LRT comparing the multivariate model containing the variable with the model that did not. The family history analysis was repeated stratifying by sex, since sex differences have been reported.[10]

Using the pedigrees, the total numbers of affected and unaffected first and second- degree relatives were determined. The ratio of affected to total relatives was calculated overall and by sex of the relative, proband, and relative and proband. Associations were assessed using the chi-square test.

## Results

Of 1504 invited families, 827 completed questionnaires (participation rate = 55%). 42 families were excluded because the foot condition was not idiopathic CTEV. This analysis includes 785 probands.

### Participant characteristics

The male∶female ratio was 2.3∶1 (Tables 1, 2). More than half had bilateral CTEV (58%). In unilateral cases the right foot was affected most often (56% right, 44% left). CTEV in first-second degree family members was reported by 11% of families, in first-third degree relatives by 16% and in ‘any’ family member by 26%.

**Table 1:** Study Population Characteristics by Country (part a).

Variable	Categories	Country					Total	
		*UK*		Netherlands				
		n	(%)^1^	n	(%)^1^	χ^2^/*P*	n	(%)^a^
Participants	Total	346	(44.1)	439	(55.9)	-	785	(100)
Sex of proband	Male	249	(72.0)	301	(68.6)	1.07/0.30	550	(70.1)
	Female	97	(28.0)	138	(31.4)		235	(29.9)
	Male∶Female		2.57∶1		2.18∶1			2.34∶1
Laterality of CTEV	Left	60	(17.4)	84	(19.2)	1.39/0.41	144	(18.4)
	Right	76	(20.0)	107	(24.4)		183	(23.4)
	Bilateral	209	(60.6)	247	(56.4)		456	(58.2)
	Unilateral	136	(39.4)	191	(43.6)	1.39/0.24	327	(41.2)
	Bilateral	209	(60.6)	247	(56.4)		456	(58.2)
Year of birth (proband)	1941–1980	12	(3.5)	14	(3.2)	46.40/<0.01	26	(3.31)
	1981–1990	66	(19.1)	58	(13.2)		124	(15.8)
	1991–1995	126	(36.4)	97	(22.1)		223	(28.4)
	1996–2000	120	(34.7)	182	(41.5)		302	(38.5)
	2000–2003	22	(6.4)	88	(20.0)		110	(14.0)
Birthweight (proband, grams)	<2500	18	(5.2)	33	(7.9)	3.37/0.50	51	(6.7)
	2500–2999	37	(10.1)	50	(11.9)		87	(11.4)
	3000–3499	124	(35.8)	150	(35.7)		274	(35.8)
	3500–3999	122	(35.3)	130	(31.0)		252	(32.9)
	≥4000	45	(13.0)	57	(13.6)		102	(13.3)
Gestation of pregnancy (weeks)^c^	<36	13	(3.8)	22	(5.1)	0.74/0.39	35	(4.5)
	≥36	329	(96.2)	410	(94.9)		739	(95.5)
Ethnicity of mother	White	341	(98.6)	426	(97.3)	1.53/0.22	767	(97.8)
	Other	5	(1.4)	12	(2.7)		17	(2.2)
Ethnicity of father	White	331	(96.2)	427	(97.5)	1.04/0.31	758	(96.9)
	Other	13	(3.8)	11	(2.5)		24	(3.1)
Maternal age at birth (years)^c^	≤24	28	(8.1)	22	(5.0)	6.80/0.08	50	(6.4)
	25–29	116	(33.5)	129	(29.5)		245	(31.3)
	30–34	138	(39.9)	210	(48.0)		348	(44.4)
	35+	64	(18.5)	77	(17.6)		141	(18.0)
Paternal age at birth (years)^c^	≤24	10	(2.9)	2	(0.5)	14.80/<0.01	11	(1.4)
	25–29	73	(21.4)	76	(17.3)		149	(19.1)
	30–34	127	(37.1)	208	(47.4)		335	(43.0)
	35+	132	(38.6)	152	(34.6)		284	(36.4)
Age of mother at first pregnancy (years)	≤24	99	(28.8)	71	(16.4)	19.63/<0.01	170	(21.9)
	25–29	160	(46.5)	220	(50.7)		380	(48.8)
	30–34	73	(21.2)	129	(29.7)		202	(26.0)
	35+	12	(3.5)	14	(3.2)		33	(3.3)
Rank of index pregnancy	1	144	(41.6)	214	(48.8)	4.30/0.12	358	(45.6)
	2	122	(35.3)	142	(32.4)		264	(33.6)
	3+	80	(23.1)	83	(18.9)		163	(20.7)
Total pregnancies (including index)	1	48	(13.9)	75	(17.1)	1.70/0.43	123	(15.7)
	2	140	(40.5)	177	(40.3)		317	(40.4)
	3+	158	(45.7)	187	(42.6)		345	(44.0)
Previous miscarriage	Yes	99	(28.7)	127	(28.9)	0.01/0.93	226	(28.9)
	No	246	(71.3)	311	(71.0)		557	(71.1)
Previous stillbirth	Yes	5	(1.5)	8	(1.8)	0.17/0.68	13	(1.7)
	No	341	(98.6)	430	(98.2)		771	(98.3)
Periconceptional folic acid supplementsb,c	Yes	195	(56.7)	227	(51.8)	1.83/0.18	422	(54.0)
	No	149	(43.3)	211	(48.2)		360	(46.0)
Oral contraceptives early pregnancyb,c	Yes	12	(3.9)	3	(0.7)	9.22/<0.01	15	(2.0)

a Percentages may not total 100 because of rounding.

b Maternal use/condition.

c index pregnancy.

**Table 2:** Study Population Characteristics by Country (part b).

Variable	Categories	Country					Total	
		*UK*		Netherlands				
		n	(%)^1^	n	(%)^1^	χ^2^/*P*	n	(%)^a^
	No	298	(96.1)	430	(99.3)			
Periconceptional tobacco use^b^ ^c^	Yes	64	(18.6)	107	(24.4)	3.84/0.05	171	(21.8)
	No	281	(81.5)	332	(75.6)			
Paternal periconceptional tobacco use^c^	Yes	95	(27.7)	129	(29.5)	0.31/0.58	224	(28.7)
	No	248	(72.3)	308	(70.5)			
Alcohol^b^ ^c^	Yes	177	(51.3)	131	(29.9)	37.02/<0.01	308	(39.3)
	No	168	(48.7)	307	(70.1)			
Maternal diabetes^c^	Yes	4	(1.2)	10	(2.3)	1.39/0.24	14	(1.8)
	No	342	(98.8)	429	(97.7)		771	(98.2)
Maternal epilepsy^c^	Yes	3	(0.9)	3	(0.7)	0.08/0.77	6	(0.8)
	No	343	(99.1)	435	(99.3)		778	(99.2)
Maternal infection (any)^c^	Yes	42	(13.1)	51	(11.6)	0.37/0.54	93	(12.2)
	No	279	(86.9)	388	(88.4)		667	(87.8)
Pre-eclampsia^c^	Yes	19	(5.5)	28	(6.4)	0.27/0.60	47	(6.0)
	No	325	(94.5)	408	(93.6)		733	(94.0)
Amniocentesis^c^	Yes	40	(11.7)	19	(4.3)	15.04/<0.01	59	(7.6)
	No	301	(88.3)	420	(95.7)		721	(92.4)
Chorionic villus sampling^c^	Yes	3	(0.9)	7	(1.6)	0.72/0.40	10	(1.3)
	No	330	(99.1)	431	(98.4)		761	(98.7)
Birth presentation (proband)	Cephalic	323	(94.7)	418	(96.3)	1.15/0.28	741	(95.6)
	Breech	18	(5.3)	16	(3.7)		34	(4.4)
Forceps delivery^c^	Yes	39	(11.3)	12	(2.7)	23.25/<0.01	51	(6.5)
	No	306	(88.7)	426	(97.3)			
Suction delivery^c^	Yes	27	(7.8)	44	(10.1)	1.15/0.28	71	(9.1)
	No	318	(92.2)	394	(90.0)			
Caesarean delivery^c^	Yes	56	(16.2)	38	(8.7)	10.43/<0.01	94	(12.0)
	No	289	(83.8)	400	(91.3)			
Multiple birth^c^	Twin^c^	7	(2.0)	16	(3.6)	1.79/0.18	23	(2.9)
	Singleton^c^	339	(98.0)	423	(96.4)		762	(97.1)
1^st^–2^nd^ degree family history of CTEV	Yes	37	(10.7)	45	(10.3)	0.04/0.84	82	(10.5)
	No	309	(89.3)	394	(89.8)		703	(89.6)
1^st^–3^rd^ degree family history of CTEV	Yes	55	(15.9)	72	(16.4)	0.04/0.85	127	(16.2)
	No	291	(84.1)	367	(83.6)		658	(83.8)
Any family history of CTEV	Yes	77	(22.3)	123	(28.0)	3.39/0.07	200	(25.5)
	No	269	(77.8)	316	(72.0)		585	(74.5)

a Percentages may not total 100 because of rounding.

b Maternal use/condition.

c index pregnancy.

### Family history associations

Factors that interacted with first-second degree family history in relation to CTEV risk were: maternal OC use, maternal use of folic acid-containing supplements, maternal ethnicity, twin birth and birthweight (Tables 3, 4, 5). Compared to those with no family history, probands with a family history were more likely to have a twin, have mothers who were non-Caucasian, and have mothers who took OCs in early pregnancy; they were less likely to have mothers who took folic acid supplements periconceptionally.

**Table 3:** Association Between Epidemiological Variables and 1^st^–2^nd^ Degree Family History (part a).

		1^st^–2^nd^ degree family history				Minimally adjusted^a^		LRT	Multivariate^b^		LRT
		Yes		No							
Variable	Categories	n	(%)	n	(%)	ORca	95% CIs	χ^2^/*P*	ORca	95% CIs	χ^2^/*P*
Participants	Total	82	(10.4)	703	(89.6)						
Country	UK	37	(45.1)	309	(43.9)	1.00	reference	0.04/0.84	1.00	reference	0.19/0.66
	Netherlands	45	(54.9)	394	(56.1)	0.95	[0.60, 1.51]		0.90	[0.55, 1.47]	
Sex	Male	51	(62.2)	499	(71.0)	1.00		2.57/0.25	1.00	reference	2.29/0.13
	Female	31	(37.8)	204	(29.0)	1.49	[0.93, 2.40]		1.49	[0.90, 2.48]	
	Male∶female		1.65∶1		2.45∶1	-	-		-	-	
Laterality of CTEV	Left	15	(18.3)	129	(18.4)	1.00	reference	3.33/1.90	1.00	reference	2.68/0.26
	Right	13	(15.9)	170	(24.3)	0.66	[0.30, 1.43]		0.70	[0.31, 1.59]	
	Bilateral	54	(65.9)	402	(57.4)	1.15	[0.63, 0.21]		1.19	[0.61, 2.29]	
	Unilateral	28	(34.2)	299	(42.7)	1.00	reference	2.21/0.14	1.00	reference	1.97/0.16
	Bilateral	54	(65.9)	402	(57.4)	1.43	[0.89, 2.32]		1.43	[0.86, 2.39]	
Year of birth (proband)	1941–1980	5	(6.1)	21	(3.0)	2.43	[0.85,6.95]	4.82/0.44	2.07	[0.60,7.17]	2.33/0.68
	1981–1990	18	(22.0)	106	(15.1)	1.73	[0.91,3.28]		1.60	[0.75,3.40]	
	1991–1995	22	(26.9)	201	(28.6)	1.12	[0.61,2.03]		1.11	[0.56,2.19]	
	1996–2000	27	(32.9)	275	(39.1)	1.00	reference		1.00	reference	
	2000–2003	10	(12.2)	100	(14.2)	1.02	[0.47,2.19]		1.16	[0.52,2.60]	
Birthweight (proband, grams)	<2500	7	(8.9)	44	(6.4)	1.53	[0.63, 3.76]	9.06/0.06	1.20	[0.50, 3.14]	8.95/0.06
	2500–2999	10	(12.7)	77	(11.2)	1.24	[0.57, 2.69]		1.19	[0.55, 2.59]	
	3000–3499	26	(32.9)	248	(36.1)	1.00	reference		1.00	reference	
	3500–3999	18	(22.8)	234	(34.1)	0.73	[0.39, 1.37]		0.58	[0.30, 1.12]	
	≥4000	18	(22.8)	84	(12.2)	2.05	[1.07, 3.92]		1.70	[0.87, 3.32]	
Gestation of pregnancy (weeks)^c^	<36	5	(6.3)	30	(4.3)	1.00	reference	0.57/0.45	1.00	reference	0.53/0.47
	≥36	75	(93.8)	664	(95.7)	0.68	[0.25, 1.80]		0.64	[0.19, 2.10]	
Ethnicity of mother	White	78	(95.1)	689	(98.2)	1.00	reference	2.50/0.11	1.00	reference	4.02/0.05
	Other	4	(4.9)	13	(1.9)	2.74	[0.87, 8.66]		3.94	[1.17, 13.32]	
Ethnicity of father	White	78	(96.3)	680	(97.0)	1.00	reference	0.11/0.74	1.00	reference	0.22/0.64
	Other	3	(3.7)	21	(3.0)	1.24	[0.36, 4.24]		1.37	[0.38, 4.86]	

Abbreviations:CI, confidence interval; ORca, Case-only odds ratio; LRT, likelihood ratio test.

a Adjusted for centre.

b Adjusted for centre, birthweight, maternal use of supplements containing folic acid (during the three months before the pregnancy or during the first trimester), and use of oral contraceptives when the mother recognised the pregnancy.

c Index pregnancy.

**Table 4:** Association Between Epidemiological Variables and 1^st^–2^nd^ Degree Family History (part b).

		1^st^–2^nd^ degree family history				Minimally adjusted^a^		LRT	Multivariate^b^		LRT
		Yes		No							
Variable	Categories	n	(%)	n	(%)	ORca	95% CIs	χ^2^/*P*	ORca	95% CIs	χ^2^/*P*
Maternal age at birth (years)^c^	≤24	8	(9.8)	42	(6.0)	1.00	reference	2.58/0.46	1.00	reference	0.51/0.92
	25–29	28	(34.2)	217	(30.9)	0.68	[0.29, 1.59]		0.82	[0.30–2.23]	
	30–34	31	(37.8)	317	(45.2)	0.51	[0.22, 1.20]		0.73	[0.27–1.98]	
	35+	15	(18.3)	126	(18.0)	0.63	[0.25, 1.58]		0.86	[0.30–2.52]	
Paternal age at birth (years)^c^	≤24	2	(2.5)	10	(1.4)	1.00	reference	1.07/0.79	1.00	reference	0.87/0.83
	25–29	18	(22.2)	131	(18.7)	0.70	[0.14, 3.48]		0.58	[0.11, 3.11]	
	30–34	32	(39.5)	303	(43.4)	0.54	[0.11, 2.61]		0.53	[0.10, 2.75]	
	35+	29	(35.8)	255	(36.5)	0.58	[0.12, 2.80]		0.64	[0.12, 3.36]	
Age of mother at first pregnancy (years)	≤24	21	(25.6)	149	(21.2)	1.00	reference	0.91/0.92	1.00	reference	0.20/0.98
	25–29	37	(45.1)	343	(48.8)	0.77	[0.43, 1.36]		0.99	[0.52, 1.90]	
	30–34	21	(25.6)	181	(25.8)	0.83	[0.43, 1.59]		1.13	[0.54, 2.35]	
	35+	3	(3.7)	30	(4.3)	0.71	[0.20, 2.54]		1.06	[0.28, 3.99]	
Rank of index pregnancy	1	44	(53.7)	314	(44.7)	1.00	reference	2.51/0.47	1.00	reference	3.64/0.16
	2	23	(28.1)	241	(34.3)	0.68	[0.40, 1.16]		0.60	[0.33, 1.07]	
	3+	15	(18.3)	148	(21.0)	0.72	[0.39, 1.34]		0.65	[0.33, 1.27]	
Total pregnancies (including proband)	1	10	(12.2)	113	(16.1)	1.00	reference	1.29/0.73	1.00	reference	1.41/0.49
	2	32	(39.0)	285	(40.5)	1.27	[0.60, 2.66]		1.55	[0.69, 3.48]	
	3+	40	(48.8)	305	(43.4)	1.48	[0.72, 3.06]		1.56	[0.71, 3.45]	
Previous miscarriage	Yes	23	(28.1)	203	(29.0)	0.96	[0.58, 1.59]	0.03/0.86	0.85	[0.47, 1.46]	0.37/0.54
	No	59	(72.0)	498	(71.0)	1.00	reference		1.00	reference	
Previous stillbirth	Yes	0	(0.0)	13	(1.9)	-	-	-	-	-	-
	No	82	(100.0)	689	(98.2)	-	-	-	-	-	
Periconceptional folic acid supplements^c^ ^d^	Yes	36	(43.9)	386	(55.0)	0.64	0.40, 1.01]	3.77/0.05	0.62	[0.38, 1.01]	3.71/0.05
	No	46	(56.1)	314	(44.9)	1.00	reference		1.00	reference	
Oral contraceptives early pregnancy^c^ ^d^	Yes	4	(5.1)	11	(1.7)	3.17	[0.97, 10.38]	3.05/0.08	3.21	[0.94, 10.99]	2.94/0.09
	No	74	(94.9)	654	(98.4)	1.00	reference		1.00	reference	

Abbreviations:CI, confidence interval; ORca, Case-only odds ratio; LRT, likelihood ratio test.

a Adjusted for centre.

b Adjusted for centre, birthweight, maternal use of supplements containing folic acid (during the three months before the pregnancy or during the first trimester), and use of oral contraceptives when the mother recognised the pregnancy.

c Index pregnancy.

d maternal use.

**Table 5:** Association Between Epidemiological Variables and 1^st^–2^nd^ Degree Family History (part c).

		1^st^–2^nd^ degree family history				Minimally adjusted^a^		LRT	Multivariate^b^		LRT
		Yes		No							
Variable	Categories	n	(%)	n	(%)	ORca	95% CIs	χ^2^/*P*	ORca	95% CIs	χ^2^/*P*
Periconceptional tobacco use^c^ ^d^	Yes	14	(17.1)	157	(22.4)	0.72	[0.39, 1.31]	1.24/0.27	0.64	[0.34, 1.22]	2.00/0.16
	No	68	(82.9)	545	(77.6)	1.00	reference		1.00	reference	
Paternal periconceptional tobacco use^c^	Yes	22	(27.5)	202	(28.9)	0.94	[0.56, 1.57]	0.06/0.80	0.81	[0.46, 1.43]	0.52/0.47
	No	58	(72.5)	498	(71.1)	1.00	reference		1.00	reference	
Alcohol^c^ ^d^	Yes	24	(29.3)	284	(40.5)	0.59	[0.35, 0.98]	4.41/0.04	0.68	[0.40, 1.16]	2.07/0.15
	No	58	(70.7)	417	(59.5)	1.00	reference		1.00	reference	
Maternal diabetes^c^	Yes	1	(1.2)	13	(1.9)	0.66	[0.09, 5.12]	0.18/0.67	-	-	-
	No	81	(98.8)	690	(98.2)	1.00	reference		-	-	
Maternal epilepsy^c^	Yes	0	(0.0)	6	(0.9)	-	-	-	-	-	-
	No	82	(100.0)	696	(99.2)	-	-		-	-	
Maternal infection (any)^c^	Yes	6	(7.6)	87	(12.8)	0.56	[0.24, 1.33]	1.99/0.16	0.53	[0.21, 1.37]	2.01/0.16
	No	73	(92.4)	594	(87.2)	1.00	reference		1.00	reference	
Pre-eclampsia^c^	Yes	6	(7.4)	41	(5.9)	1.29	[0.53, 3.13]	0.29/0.59	1.45	[0.57, 3.64]	0.57/0.45
	No	75	(92.6)	658	(94.1)	1.00	reference		1.00	reference	
Amniocentesis^c^	Yes	9	(11.0)	50	(7.2)	1.59	[0.74, 3.39]	1.31/0.25	1.96	[0.89, 4.30]	2.50/0.11
	No	73	(89.0)	648	(92.8)	1.00	reference		1.00	reference	
Chorionic villus sampling^c^	Yes	0	(0.0)	10	(1.5)	-	-		-	-	
	No	81	(100.0)	680	(98.6)	-	-		-	-	
Birth presentation (proband)	Breech	2	(2.5)	32	(4.6)	0.53	[0.12, 2.25]	0.89/0.35	0.56	[0.13, 2.40]	0.72/0.40
	Cephalic	78	(97.5)	663	(95.4)	1.00	reference		1.00	reference	
Forceps delivery^c^	Yes	8	(9.8)	43	(6.1)	1.66	[0.74, 3.72]	1.37/0.24	1.34	[0.55, 3.27]	0.40/0.53
	No	74	(90.2)	658	(93.9)	1.00	reference		1.00	reference	
Suction delivery^c^	Yes	7	(8.5)	64	(9.1)	0.93	[0.41, 2.11]	0.03/0.86	1.09	[0.47, 2.54]	0.04/0.84
	No	75	(91.5)	637	(90.9)	1.00	reference		1.00	reference	
Caesarean delivery^c^	Yes	7	(8.5)	87	(12.4)	0.65	[0.29, 1.46]	1.20/0.27	0.75	[0.33, 1.71]	0.50/0.48
	No	75	(91.5)	614	(87.6)	1.00	reference		1.00	reference	
Multiple birth^c^	Twin	5	(6.1)	18	(2.6)	2.50	[0.90, 6.93]	2.61/0.11	3.87	[1.19, 12.62]	4.37/0.04
	Singleton	77	(93.9)	685	(97.4)	1.00	reference		1.00	reference	

Abbreviations:CI, confidence interval; ORca, Case-only odds ratio; LRT, likelihood ratio test.

a Adjusted for centre.

b Adjusted for centre, birthweight, maternal use of supplements containing folic acid (during the three months before the pregnancy or during the first trimester), and use of oral contraceptives when the mother recognised the pregnancy.

c Index pregnancy.

d maternal use.

Maternal smoking in the periconceptional period was less common in those with a family history, reflected in an inverse, but non-statistically significant, ORca (multivariate ORca = 0.64, 95%CI 0.34–1.22, p = 0.16). The risk estimates were similar for smoking in the three months pre-conception and in the first trimester (data not shown). There was no association with paternal smoking.

After stratifying by sex, males with a family history were more likely than those without to have mothers who took OCs in early pregnancy (multivariate ORca = 4.35, 95%CI 1.01–18.78, p = 0.07) and to have a twin (ORca = 5.28, 95%CI 1.31–21.32, p* = *0.03), and less likely to have mothers who took folic acid-containing supplements first trimester (ORca = 0.59, 95%CI 0.31–1.10, p* = *0.10) or who had previously had a miscarriage (ORca = 0.53, 95%CI 0.24–1.17, p* = *0.10). Birthweight distribution varied between males with and without a family history (<2500 g ORca = 0.66 95%CI 0.15–2.89; 2500–2999 g ORca = 1.12, 95%CI 0.42–2.99; 3000–3499 g ORca = 1.00 [reference]; 3500–3999 g ORca = 0.43, 95%CI 0.18–1.03; ≥4000 g ORca = 1.57 95%CI 0.70–3.55; p* = *0.07). Females with a family history were less likely than those without to have been delivered by caesarean section (ORca = 0.23, 95%CI 0.03–1.86, p* = *0.10) and to have mothers who consumed alcohol (ORca = 0.33, 95%CI 0.12–0.89, p* = *0.02) or had an infection (ORca = 0.11, 95%CI 0.01–0.95, p* = *0.01) during pregnancy. They were more likely to have mothers who were non-Caucasian (ORca = 16.18, 95%CI 1.19–220.5, p* = *0.03) and who had an amniocentesis in the index pregnancy (ORca = 5.69, 95%CI 1.46–22.15, p* = *0.02).

### Associations by proband sex

The factors which interacted with sex to affect CTEV risk were: maternal gravidity and miscarriage history, chorionic villus sampling in the index pregnancy, forceps delivery, birthweight, and proband birth year. Compared to males, females were more likely to have mothers who were multiparous (two pregnancies: multivariate ORca = 2.46, 95%CI 1.40–4.30; ≥three pregnancies: ORca = 1.98, 95%CI 1.08–3.66, p* = *0.005), had a history of miscarriage (ORca = 1.42, 95%CI 0.94–2.12, p* = *0.09), and had chorionic villus sampling in the index pregnancy (ORca = 3.27, 95%CI 0.90–11.90, p* = *0.07). Females were less likely to have been delivered by forceps (ORca = 0.31, 95%CI 0.13–0.77, p* = *0.01), were lighter at birth and were more likely to be born in earlier years (data not shown).

### Associations by CTEV laterality

The factors which interacted with laterality to affect CTEV risk were: gestation, maternal gravidity and alcohol consumption, and family history. Compared to unilateral CTEV, probands affected bilaterally were less likely to have been premature (multivariate ORca = 0.51, 95%CI 0.24–1.09, p* = *0.07) and to have mothers who consumed alcohol during pregnancy (ORca = 0.76, 95%CI 0.56–1.03, p* = *0.07), but more likely to have a first- third degree family history (ORca = 1.43, 95%CI 0.95–2.14, p* = *0.08) and to have mothers who had two pregnancies in total (one pregnancy ORca 1.00 [reference], two pregnancies ORca = 1.38, 95%CI 0.89–2.12; ≥three pregnancies ORca = 0.90, 95%CI 0.59–1.39; p* = *0.03).

### Pedigree analysis

CTEV in first-degree relatives was reported in 5.7% (45/785) of families; 5.7% (45/785) had affected second-degree relatives, 1.0% (8/785) had affected first *and* second-degree relatives and 10.5% (82/785) had affected first *or* second-degree relatives. Of those with a first-degree family history, 38 had one affected relative (15 sibs, 14 fathers, nine mothers), six had two affected relatives (three sib/mother-pairs, one sib/father-pair and two sib-pairs) and one had three affected relatives (mother and two sibs). Regardless of degree of relatedness, 139 families reported one affected relative, 46 reported two, 13 reported three, two reported four and five reported five.

CTEV risk to any first-degree relative was 2.2% and to any first or second-degree relative 1.2% (Table 6). Male relatives were affected more often than female relatives (first-second degree 1.4% vs 1.0%, p* = *0.05. Table 7) and relatives of female probands were affected more often than relatives of male probands (first-second degree 1.6% vs 1.0%, p* = *0.01, Table 8). Male relatives of female probands had the highest absolute risk (first-second degree 2.0%, p* = *0.02, Table 9).

**Table 6:** Overall Risk of CTEV in 1^st^ and 2^nd^ Degree Relatives of Probands.

Relation degree	No. relatives/total relatives (%)	95% CI
1^st^ degree	53/2388 (2.2)	1.67, 2.89
1^st^–2^nd^ degree	106/9087(1.2)	0.96, 1.41

Abbreviations: CI, confidence interval.

**Table 7:** Risk of CTEV in 1^st^ and 2^nd^ Degree Relatives of Probands by the Sex of the Relative.

Relation degree	Sex of relative	No. relatives/total relatives (%)	95% CI	χ^2^/*P*
1^st^ degree	Female	23/1189 (1.9)	1.23, 2.89	0.72/0.40
	Male	29/1187 (2.4)	1.64, 3.49	
1^st^–2^nd^ degree	Female	42/4498 (1.0)	0.67, 1.26	3.88/0.05
	Male	63/4578 (1.4)	1.11, 1.76	

Abbreviations: CI, confidence interval.

**Table 8:** Risk of CTEV in 1^st^ and 2^nd^ Degree Relatives of Probands by the Sex of the Proband.

Relation degree	Sex of proband	No. relatives/total relatives (%)	95% CI	χ^2^/*P*
1^st^ degree	Female	22/719 (3.1)	1.93, 4.60	3.35/0.07
	Male	31/1669 (1.9)	1.27, 2.62	
1^st^–2^nd^ degree	Female	46/2811 (1.6)	1.20, 2.18	7.80/0.01
	Male	60/6276 (1.0)	0.73, 1.23	

Abbreviations: CI, confidence interval.

**Table 9:** Risk of CTEV in 1^st^ and 2^nd^ Degree Relatives of Probands by the Sex of the Proband and Sex of the Relative.

Relation degree	Sex of proband	Sex of relative	No. relatives/total relatives (%)	95% CI	χ^2^/*P*
1st degree	female	Female	9/348 (2.6)	1.19, 4.85	0.30/0.58
		Male	12/366 (3.3)	1.71, 5.66	
1^st^–2^nd^ degree	female	Female	17/1399 (1.2)	0.71, 1.94	2.67/0.10
		Male	28/1407 (2.0)	1.33, 2.86	
1st degree	male	Female	14/845 (1.7)	0.91, 2.86	0.39/0.53
		Male	17/821 (2.1)	1.21, 3.29	
1^st^–2^nd^ degree	male	Female	25/3103 (0.8)	0.52, 1.19	1.47/0.23
		Male	35/3170 (1.1)	0.77, 1.53	

Abbreviations: CI, confidence interval.

## Discussion

### Strengths and limitations

Most previous CTEV studies have either been based on routine data, which gives large sample sizes but lack certainty about the diagnosis of CTEV, or on small clinical series from single centres, which may be highly selected. In addition, studies do not always distinguish clearly between syndromic and idiopathic CTEV. The current study is the largest reported series of idiopathic CTEV involving primary data collection, and we carefully reviewed questionnaires to exclude syndromic CTEV and other foot conditions. The case-only design is statistically powerful for the investigation of interactions.[17], [18] The key assumption underpinning the design is independence in the population between the stratification variable and risk factor;[19] if violated, risk estimates may be biased. We are not aware of any evidence to suggest the factors considered are not independent.

Recall accuracy and diagnostic reliability are challenges in family history analyses. We confirmed positive reports by telephone interview and additional questionnaires where possible, and restricted most analyses to first-second degree history, which may be more accurately reported.

Study participants were accrued from two national support groups, raising the possibility that they might not be representative of all idiopathic CTEV. For the results to be seriously biased, the probability of participation would need to have been associated with family history, laterality or proband sex. The sex ratio and laterality distribution mirrors patterns seen elsewhere.[1], [4], [6], [11], [20]–[27] Moreover, the proportion with a family history corresponds with the upper limit of estimates from two US series,[4], [28] is consistent with the UK Talipes series,[26] and is slightly lower that in series of 120 Scottish children.[22] This suggests our results are unlikely to be seriously biased.

### Parental smoking

Reports of associations between foot deformities, including CTEV, and maternal smoking during pregnancy are inconsistent.[5], [7], [10], [29]–[33] One US case-control study of idiopathic CTEV reported a greater than multiplicative interaction between smoking and family history, such that maternal smoking increased risk only in children with a family history (OR 20.30, 95%CI 7.90–52.17).[10] We, in contrast, found no evidence of any interaction between family history and maternal (or paternal) smoking in the three months before, or first trimester of, the index pregnancy. If anything our risk estimates suggested a less than multiplicative interaction, although they were not statistically significant.

In our study maternal smoking prevalence in the first trimester was 15% (22% in the three months before the pregnancy or first trimester) compared with 38% in the first trimester among cases in the US study. This difference could be due to differences in data collection methods (interview versus postal questionnaire), study location or subjects' period of birth (1968–1980 vs 1941–2003 [>70% 1991–2000]). The US study defined family history as ‘probable’ CTEV in first-degree relatives, but when we restricted our analysis to first-degree relatives and first trimester smoking the risk estimate was further from unity (multivariate ORca = 0.59, 95%CI 0.21–1.69, p = 0.30). The CTEV-smoking relationship, in those with or without a family history, thus remains controversial, and a role for smoking in CTEV cannot be entirely ruled out.

### Perinatal factors and other maternal exposures during index pregnancy

The observed significant (p = 0.04) interaction between a positive family history and twin births is novel and may have become evident because, unlike previous studies of CTEV and twinning,[4], [34] we stratified by family history. It could be interpreted as consistent with the uterine constraint hypothesis for CTEV.[3]

As with other congenital anomalies,[35] there is some evidence of a role for folate metabolism in CTEV.[13], [36], [37] The borderline significant interaction between family history and maternal folic acid supplement use (p = 0.09) provides some further support for this. Although recall accuracy might be a concern, it seems unlikely this would be differential by family history. Since our results suggest supplement use might be associated with reduced CTEV risk in those without a family history further investigation is warranted.

Although observed in a subgroup analysis, the significant interaction (p = 0.02) between maternal alcohol consumption and family history in females is intriguing (mothers of female probands with a family history were less likely to report alcohol consumption). It is unlikely the finding reflects avoidance of ‘risky’ behaviour during pregnancy in women aware of a family history, as the association was not seen in males. Although alcohol is teratogenic,[38] it has rarely been considered in relation to CTEV and further investigation would be valuable.

The suggestion of an interaction between family history and maternal OC use in early pregnancy is of interest, especially as the effect was strongest in males. Increased risk of congenital limb deficiencies in offspring of mothers who had taken relatively high-dose OCs in the periconceptional period has been reported,[39] suggesting our finding could be due to specific OC types (e.g. higher-dose or anti-androgenic OCs). We could not explore further as we did not have information on types of OCs used. However, while some studies report modest increased risks of birth defects, including limb deformities, with OC use,[40] the FDA concluded they were not teratogenic[41] and it is unclear how much of the maternal hormones reach the fetus and whether exogenous hormones are more likely to cross the placental barrier than endogenous (P Fowler, personal communication). Moreover, since our result was only borderline significant it may be due to chance.

### Carter effect

Our results add to growing evidence for the Carter effect and a multifactorial threshold model in CTEV. The observed higher CTEV risk in relatives of female probands is consistent both with early work from Wynne-Davis et al, based on 144 UK cases born in 1940–1961,[8] and a recent US study which described increased CTEV transmission from mothers to their offspring compared with fathers.[9] Although other studies found CTEV risk was independent of the proband's sex, these included relatively few pedigrees (n<175).[4], [26] The somewhat different risk factor pattern in females and males also points towards the possibility that a higher “load” of risk factors (whether genetic and/or environmental) in families of affected girls might predispose to CTEV.

### Conclusions

Using the largest series of idiopathic CTEV with primary data collection so far reported, we set out to (1) follow-up previous observations suggesting the possibility of risk factor heterogeneity and (2) generate hypotheses for future study. Our results provide support for the ‘Carter effect’, suggesting that females require a higher risk factor ‘load’ before developing CTEV. Beyond this, although we found only tentative evidence for aetiologically distinct subgroups, our results do suggest some areas worth further exploration, including the relationships between family history and twinning and maternal use of folic acid supplements and alcohol during the index pregnancy. Large multi-centre studies, with sufficient power to fully explore risk factors in different case sub-groups, are needed to further elucidate the aetiology of this common, but poorly understood, condition.
